# T5224, RSPO2 and AZD5363 are novel drugs against functional pituitary adenoma

**DOI:** 10.18632/aging.102372

**Published:** 2019-10-26

**Authors:** Sheng Zhong, Bo Wu, Jiahui Li, Xinhui Wang, Shanshan Jiang, Fangfei Hu, Gaojing Dou, Yuan Zhang, Chunjia Sheng, Gang Zhao, Yunqian Li, Yong Chen

**Affiliations:** 1 Department of Neurosurgery, The First Hospital of Jilin University, Changchun, China; 2 Clinical College, Jilin University, Changchun, China; 3 Pharmacy College, Jilin University, Chuangchun, China; 4 Department of Orthopaedics, The First Hospital of Jilin University, Changchun, China; 5 Department of Oncology, The First Hospital of Jilin University, Changchun, China

**Keywords:** bioinformatics, brain science, drug treatment, functional pituitary adenomas, prognosis

## Abstract

We tested whether the drugs T5224, RSPO2, and AZD5363 exert therapeutic effects against functioning pituitary adenoma (FPA). We analysed the gene expression profiles of four FPA mRNA microarray datasets (GSE2175, GSE26966, GSE36314, and GSE37153) from the Gene Expression Omnibus database and identified genes differentially expressed in FPA vs control tissues. We then carried out Gene Ontology, Kyoto Encyclopedia of Genes and Genomes (KEGG), and protein-protein interaction network analyses. We also measured the difference in expression of hub genes between human normal pituitary cells and FPA cells using qRT-PCR. Our *in vitro* colony-formation and MTT assays showed that cell viability, number, and the size of clonogenicities were all lower in the presence of T5224, RSPO2, or AZD536 than in controls. Moreover, flow cytometry experiments showed that the incidence of apoptosis was higher in the presence of T5224, RSPO2, or AZD5363 than among controls, and was increased by increasing the doses of the drugs. This suggests these drugs could be used as therapeutic agents to treat FPA. Finally, we found that cFos, WNT5A, NCAM1, JUP, AKT3, and ADCY1 are abnormally expressed in FPA cells compared to controls, which highlights these genes as potential prognostic and/or therapeutic targets.

## INTRODUCTION

Functioning Pituitary Adenoma (FPA) accounts for 70% of all pituitary adenomas (PA) [1]. Epidemiological studies have suggested that the incidence and prevalence of pituitary neoplasm might be underestimated at 7.39/100,000/year and 97.76/100,000, respectively [2]. FPA can cause hyper-secretion syndromes, such as hyperprolactinemia, acromegaly, and Cushing disease, or mass effects, such as headaches, hypopituitarism, vomiting, and visual field defects [4]. Based on hormonal activity, FPA is clinically classified mainly as prolactinoma (PRL), growth hormone (GH) tumors, or adrenocorticotropic (ACTH) hormone tumors. Gonadotropin hormone tumors, multiple hormone adenomas, and thyroid-stimulating hormone (TSH) tumors occur only rarely in FPA [3]. The fact that FPA can arise from a wide variety of cancer types makes it complicated to conduct research on FPA’s diagnosis, underlying molecular mechanisms, and treatment.

Currently, early diagnosis and treatment have improved, owing to the widespread use of magnetic resonance imaging (MRI) [2]. However, diagnostic methods, such as measuring hormone levels, MRI, pathological and immunohistochemical assays, etc., are still not accurate nor timely enough to prevent morbidity and consequent mortality due to FPA. Traditional treatments such as Dopamine agonists, surgery, and radiotherapy have limited effectiveness and cause deleterious side effects, including a reduced quality of life in the presence of persistent morbidity and slightly increased mortality [4]. Many types of FPA, especially macroadenomas, have extremely low cure rates [6]. For example, in ~20% of prolactinoma cases, treatment is partially or completely ineffective [5].

Previous studies have demonstrated overexpression of high mobility group A (HMGA) in FPA, possibly due to downregulation of HMGA-targeting microRNAs (miRNAs) [7]. For example, HMGA2 is overexpressed in prolactinoma. In addition, the majority of adenomas show reduced EFEMP1 expression, irrespective of subtype [8]. Other factors like reduced expression of bone morphogenetic proteins (BMP) can cause some adenoma subtypes [9]. In this study, c-Fos, Wnt5A, and Akt3 was identified as hub genes, which could be used to treat FPA. c-Fos is component of AP-1 transcription factors, and T5224 has been reported selectively inhibit AP-1. This drug already be used in phase II human clinical trials in Japan [10]. RSPO2 can block binding of Wnt5A to Fzd7 receptor to antagonize tumor cell migration [49]. AZD5363 is one of Akt3 inhibitors and an apoptosis promoter in prostate cancer [50].

Here, we hypothesized that T5224, RSPO2, and AZD5363 should be effective treatments against FPA. To test this hypothesis, we looked for genes differentially expressed in FPA tissues compared to normal brain controls. We also performed bioinformatics analyses to investigate the molecular processes underlying FPA and used various biochemical and cell biology assays to test the effects of T5224, RSPO2, and AZD5363 treatments.

## RESULTS

### Identification of differentially expressed genes

**Figure 1:**
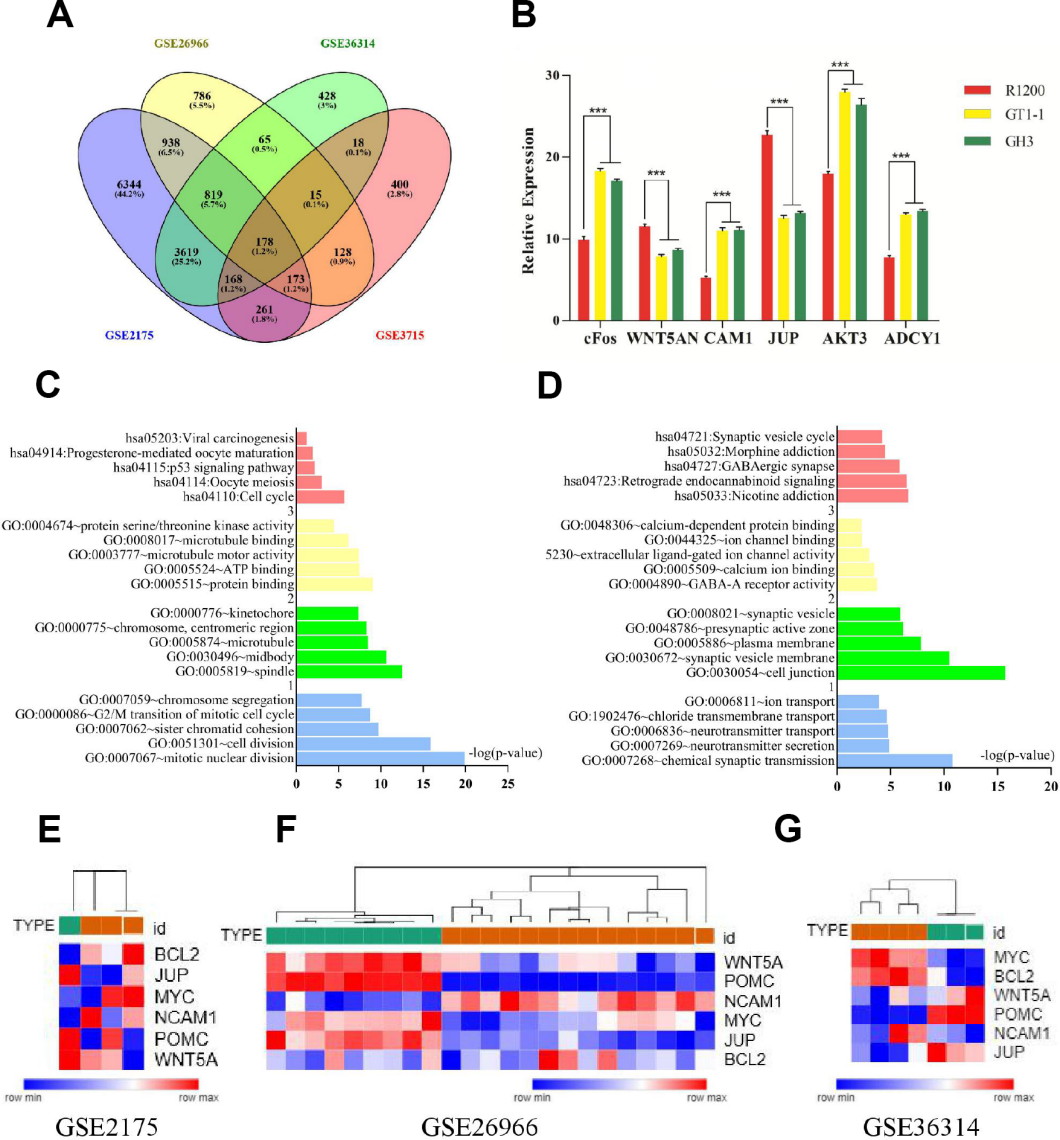
(**A**) Venn diagrams for DEGs. (**B**) Results of q-PCR analysis. (**C**) Functional and pathway enrichment analysis of up- regulated genes. (**B**) Expression heat map of hub genes. (**D**) Functional and pathway enrichment analysis of down-regulated genes. (**E**–**G**) Expression heat map of hub genes.

We analyzed the gene expression profiles of four FPA mRNA microarray datasets (GSE2175, GSE26966, GSE36314, and GSE37153) from the Gene Expression Omnibus database. There were 19,943 differently expressed genes (DEGs) picked up from GSE2175, of which 12,268 were upregulated while 7675 were downregulated. Altogether, 4635 DEGs were found out from GSE26966, among which 2159 were upregulated and 2476 were downregulated. Among 6472 DEGs were identified from GSE36314 with 2520 upregulated genes and 3952 downregulated genes. Lastly, 2020 DEGs were discovered from GSE37153, in which 2020 genes were upregulated and 1017 were downregulated. There were 178 mutual DEGs among the four datasets (Figure 1A, Supplementary Table 1).

### Functional and pathway enrichment analysis

The mutual DEGs were uploaded to DAVID for GO and KEGG pathway analyses (Table 1 and Figure 1C, 1D). The GO analysis results revealed that the mutually upregulated DEGs were mainly associated with several biological processes (BPs), such as mitotic nuclear division, cell division, and chromosome segregation; cellular components (CCs; spindle, microtubule, kinetochore); and molecular functions (MFs; protein binding, ATP binding, microtubule motor activity). For the mutually downregulated DEGs, the GO analyses revealed that they were primarily involved in BPs such as neurotransmitter secretion, neurotransmitter transport, and ion transport; CCs covering cell junctions and plasma membrane; and MFs including calcium ion binding and calcium-dependent protein binding. In addition, KEGG analyses indicated that the mutual DEGs were mainly involved in cell cycle, oocyte meiosis, and p53 signaling pathways, nicotine addiction, GABAergic synapse, and morphine addiction.

**Table 1:** Functional and pathway enrichment analysis of up-regulated and down-regulated genes among four datasets.

**Expression**	**Category**	**Term**	**Count**	**%**	**P Value**
up-regulated	GOTERM_BP_DIRECT	GO:0007067~mitotic nuclear division	18	38.29787234	1.28E-20
	GOTERM_BP_DIRECT	GO:0051301~cell division	17	36.17021277	1.39E-16
	GOTERM_BP_DIRECT	GO:0007062~sister chromatid cohesion	9	19.14893617	1.85E-10
	GOTERM_BP_DIRECT	GO:0000086~G2/M transition of mitotic cell cycle	9	19.14893617	1.82E-09
	GOTERM_BP_DIRECT	GO:0007059~chromosome segregation	7	14.89361702	1.91E-08
	GOTERM_CC_DIRECT	GO:0005819~spindle	11	23.40425532	2.99E-13
	GOTERM_CC_DIRECT	GO:0030496~midbody	10	21.27659574	2.40E-11
	GOTERM_CC_DIRECT	GO:0005874~microtubule	11	23.40425532	3.41E-09
	GOTERM_CC_DIRECT	GO:0000775~chromosome, centromeric region	7	14.89361702	5.29E-09
	GOTERM_CC_DIRECT	GO:0000776~kinetochore	7	14.89361702	4.53E-08
	GOTERM_MF_DIRECT	GO:0005515~protein binding	41	87.23404255	9.05E-10
	GOTERM_MF_DIRECT	GO:0005524~ATP binding	18	38.29787234	3.45E-08
	GOTERM_MF_DIRECT	GO:0003777~microtubule motor activity	7	14.89361702	4.29E-08
	GOTERM_MF_DIRECT	GO:0008017~microtubule binding	8	17.0212766	7.29E-07
	GOTERM_MF_DIRECT	GO:0004674~protein serine/threonine kinase activity	8	17.0212766	3.54E-05
	KEGG_PATHWAY	hsa04110:Cell cycle	6	12.76595745	1.97E-06
	KEGG_PATHWAY	hsa04114:Oocyte meiosis	4	8.510638298	9.73E-04
	KEGG_PATHWAY	hsa04115:p53 signaling pathway	3	6.382978723	0.00674313
	KEGG_PATHWAY	hsa04914:Progesterone-mediated oocyte maturation	3	6.382978723	0.01117001
	KEGG_PATHWAY	hsa05203:Viral carcinogenesis	3	6.382978723	0.05510903
down-regulated	GOTERM_BP_DIRECT	GO:0007268~chemical synaptic transmission	16	14.15929204	1.58E-11
	GOTERM_BP_DIRECT	GO:0007269~neurotransmitter secretion	6	5.309734513	1.41E-05
	GOTERM_BP_DIRECT	GO:0006836~neurotransmitter transport	5	4.424778761	1.73E-05
	GOTERM_BP_DIRECT	GO:1902476~chloride transmembrane transport	7	6.194690265	2.15E-05
	GOTERM_BP_DIRECT	GO:0006811~ion transport	7	6.194690265	1.24E-04
	GOTERM_CC_DIRECT	GO:0030054~cell junction	25	22.12389381	1.97E-16
	GOTERM_CC_DIRECT	GO:0030672~synaptic vesicle membrane	10	8.849557522	3.25E-11
	GOTERM_CC_DIRECT	GO:0005886~plasma membrane	52	46.01769912	1.47E-08
	GOTERM_CC_DIRECT	GO:0048786~presynaptic active zone	6	5.309734513	7.06E-07
	GOTERM_CC_DIRECT	GO:0008021~synaptic vesicle	8	7.079646018	1.22E-06
	GOTERM_MF_DIRECT	GO:0004890~GABA-A receptor activity	4	3.539823009	1.88E-04
	GOTERM_MF_DIRECT	GO:0005509~calcium ion binding	14	12.38938053	3.42E-04
	GOTERM_MF_DIRECT	GO:0005230~extracellular ligand-gated ion channel activity	4	3.539823009	9.96E-04
	GOTERM_MF_DIRECT	GO:0044325~ion channel binding	5	4.424778761	0.00472253
	GOTERM_MF_DIRECT	GO:0048306~calcium-dependent protein binding	4	3.539823009	0.00505404
	KEGG_PATHWAY	hsa05033:Nicotine addiction	7	6.194690265	2.30E-07
	KEGG_PATHWAY	hsa04723:Retrograde endocannabinoid signaling	9	7.96460177	3.09E-07
	KEGG_PATHWAY	hsa04727:GABAergic synapse	8	7.079646018	1.40E-06
	KEGG_PATHWAY	hsa05032:Morphine addiction	7	6.194690265	3.07E-05
	KEGG_PATHWAY	hsa04721:Synaptic vesicle cycle	6	5.309734513	6.13E-05

### Module screening from the PPI network

**Figure 2:**
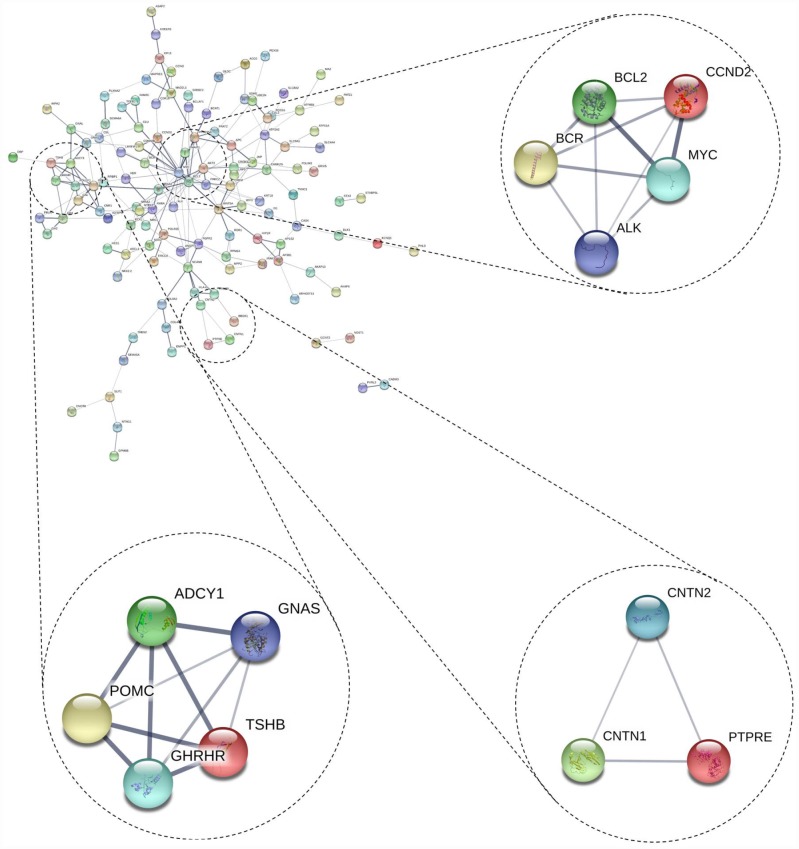
**Top 3 modules from the protein-protein interaction network.**

We also conducted PPI network analyses of the previous 178 mutual DEGs. Genes with degrees ≥ 6 were screened as hub genes based on the STRING database. Altogether, 21 genes were identified as hub genes (Figure 1E–1G), including c-Fos, MYC, BCL2, WNT5A, POMC, NCAM1, JUP, AKT3, ADCY1, FGFR2, GH1, CCND2, TSHB, GHRHR, PPP2R5A, BCR, CAMK2G, ATP2A2, APC, and MAD2L1 (listed in Table 2). MYC had the highest degree of nodes, which was 20. Moreover, after MCODE analysis, 157 nodes and 797 edges were obtained, as well as the top three modules (Figure 2), whose functional annotation and enrichment are shown in Table 3. Enriched function analysis revealed that genes in module 1 were primarily related to cell proliferation, protein complex formation, and negative regulation of apoptosis. In module 2, the genes were mainly enriched in activation of adenylate cyclase activity, adenylate cyclase-activating G-protein coupled receptor signaling pathways, and regulation of lipolysis in adipocytes. Finally, for module 3, the genes were involved in glycoprotein binding, anchored component of membrane, and myelin sheath formation.

**Table 2:** Detailed information of the hub genes among four datasets.

**Gene symbol**	**Degree**	**Betweenness centrality**	**Gene symbol**	**Degree**	**Betweenness centrality**
cFos	22	0.3840231	GH1	6	0.01536337
MYC	20	0.3150368	CCND2	6	0.02628263
WNT5A	18	0.1803458	TSHB	6	0.01756622
BCL2	17	0.37660255	GHRHR	6	0.00112801
NCAM1	13	0.24487191	PPP2R5A	6	0.11767315
JUP	12	0.0401026	BCR	6	0.03518683
POMC	11	0.05597786	CAMK2G	6	0.03972707
AKT3	10	0.03310717	ATP2A2	6	0.12136994
ADCY1	9	0.00765123	APC	5	0.02201292
FGFR2	7	0.0745872	MAD2L1	5	0.07012635

**Table 3:** Functional and pathway enrichment analysis of the modules genes.

**Module**	**Term**	**Count**	**P Value**	**FDR**	**Genes**
module 1	GO:0008283~cell proliferation (BP)	3	2.76E-03	3.48761792	BCL2, ALK, MYC
	GO:0043234~protein complex (CC)	3	2.97E-03	2.382996199	BCR, ALK, MYC
	GO:0043066~negative regulation of apoptotic process (BP)	3	4.24E-03	5.307949002	CCND2, BCL2, MYC
module 2	GO:0007190~activation of adenylate cyclase activity(BP)	3	3.31E-05	3.62E-02	ADCY1, GNAS, GHRHR
	GO:0007189~adenylate cyclase-activating G-protein coupled receptor signaling pathway(BP)	3	5.19E-05	5.67E-02	ADCY1, GNAS, GHRHR
	hsa04923:Regulation of lipolysis in adipocytes(KEGG)	3	3.83E-04	3.72E-01	ADCY1, TSHB, GNAS
module 3	GO:0001948~glycoprotein binding(MF)	2	7.69E-03	3.89981344	CNTN2, CNTN1
	GO:0031225~anchored component of membrane(CC)	2	1.24E-02	8.45522442	CNTN2, CNTN1
	GO:0043209~myelin sheath(CC)	2	1.66E-02	11.21564142	CNTN2, CNTN1

### Measuring expression of hub genes by qRT-PCR

We performed qRT-PCR in order to conform the expression of cFos, WNT5A, NCAM1, JUP, AKT3, and ADCY1 in normal pituitary cells and FPA cells (GT1-1, GH3). NCAM1, cFos, AKT3, and ADCY1 were consistently upregulated in FPA cells compared to normal pituitary cells (P < 0.05) while WNT5A and JUP were downregulated (P < 0.05)., with levels being slightly different across the tested cell lines (showed in Figure 1B).

### T5224, RSPO2, and AZD5363 reduce proliferation of FPA cells

**Figure 3:**
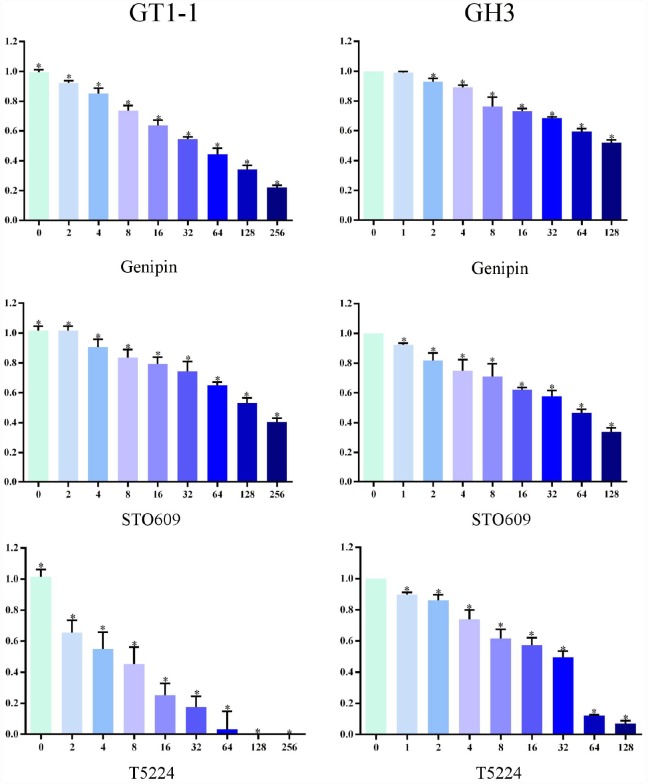
**Cellular viability of glioblastoma cells treated with T5224, Genipin and STO-609.**

**Figure 4:**
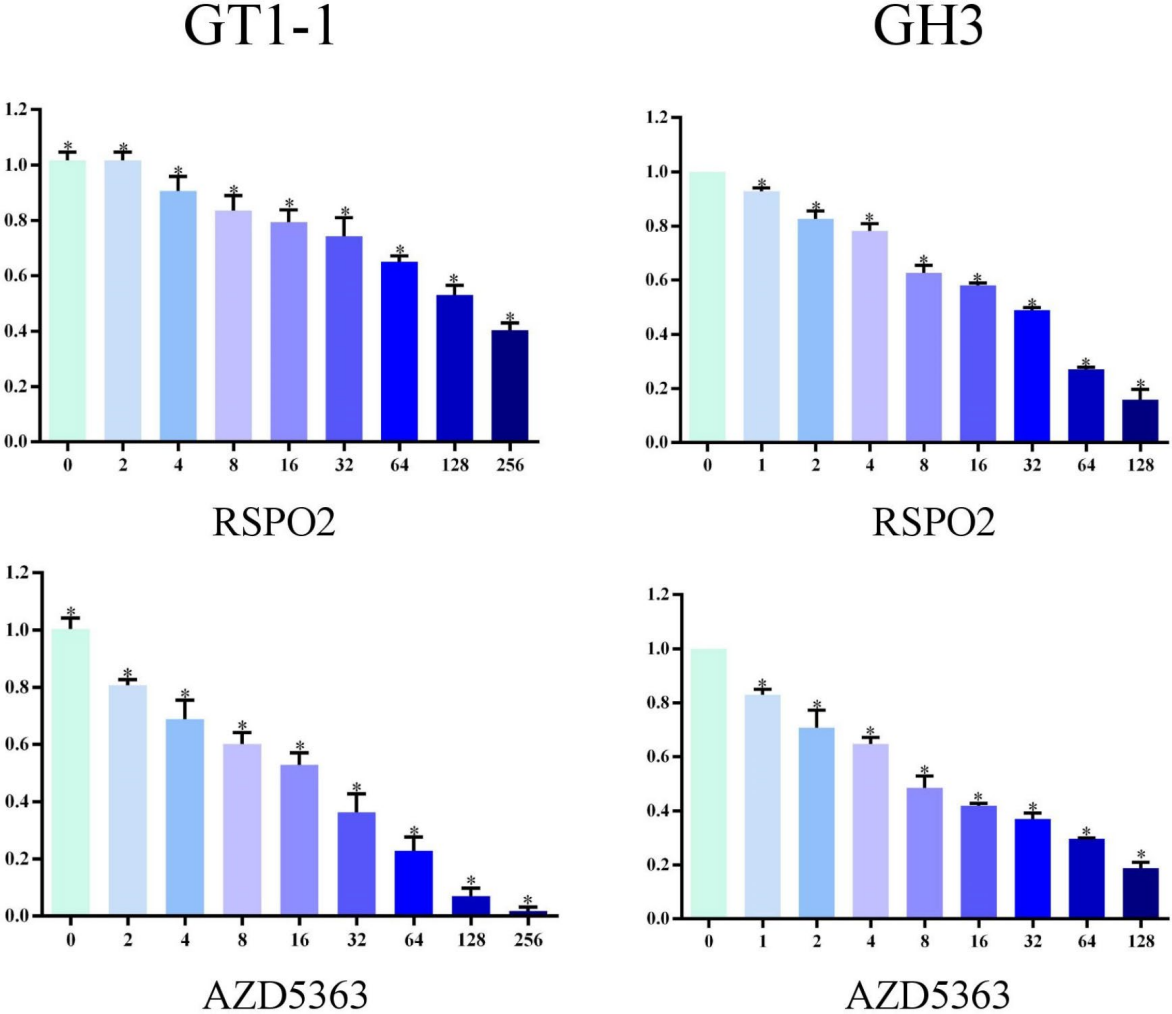
**Cellular viability of glioblastoma cells treated with RSPO2 and AZD5363.**

We used MTT assay to measure cell survival after T5224, RSPO2, and AZD5363 treatment. As is shown in Figures 3 and 4, with increasing drug concentrations, cellular viability (ratio to controls) in cell lines GT1-1 and GH3 dropped, decreasing more rapidly for T5224 than for STO609, Genipin (P < 0.05), RSPO2, and AZD5363. Besides, compared to STO609, cellular viability also declined faster for Genipin in cell line GT1-1. However, in GH3 cells, cellular viability was similar for STO609 and Genipin.

**Figure 5:**
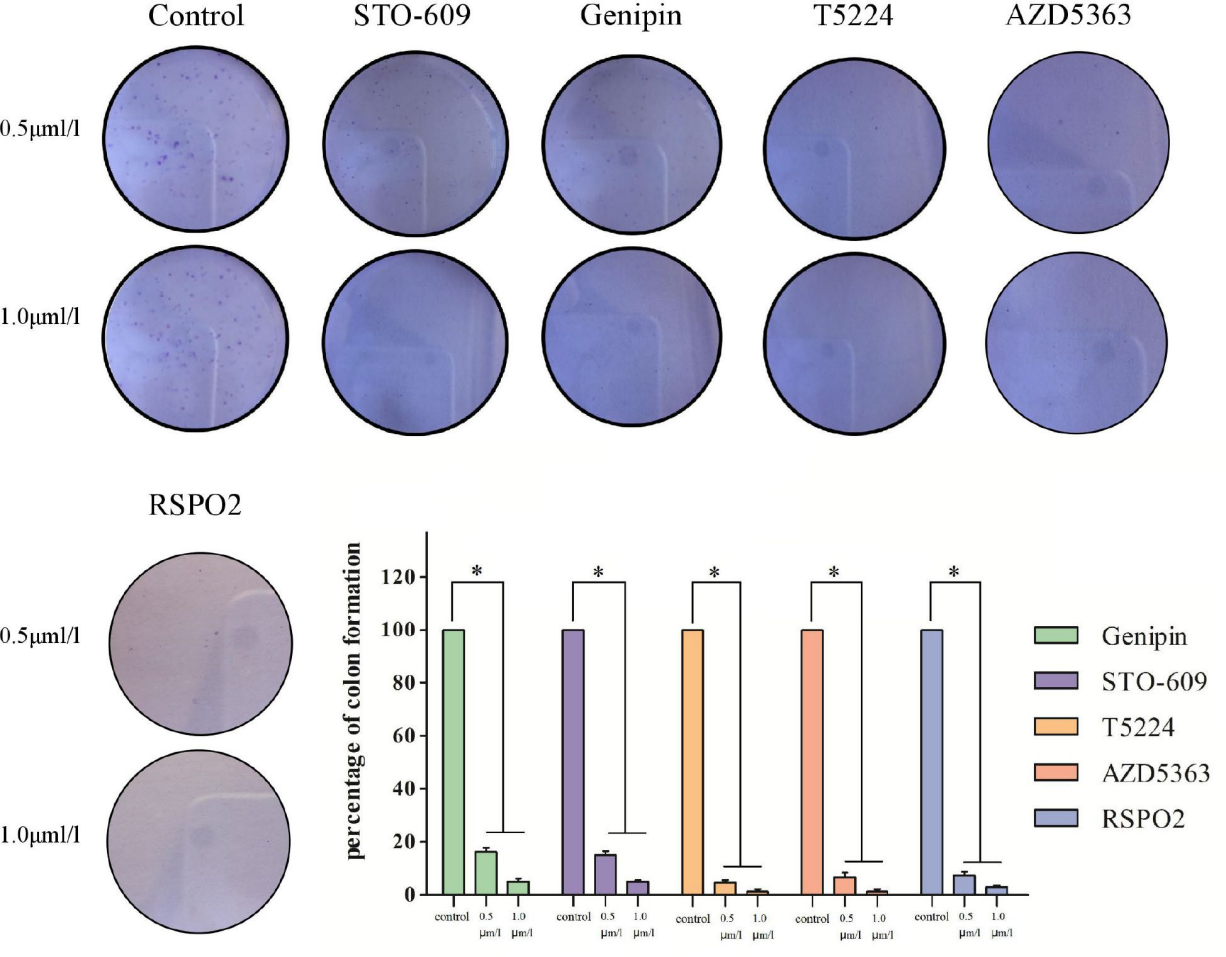
**Clonogenicities in Petri dishes with different dose of T5224, RSPO2, AZD5363, Geinpin, and STO-609.**

Colony-formation assays revealed different percentages of clone formation for each drug treatment group. Compared to controls, there were fewer and smaller colonies in all drug groups (0.5μmol/L, 1μmol/L). On the other hand, clonogenicities were approximately the same for the STO609 and Genipin groups, both of which were higher than those for the T5224, RSPO2, and AZD5363 groups (P < 0.05) (Figure 5). Higher drug concentrations correlated with fewer clonogenicities, implying dose-dependent effects for T5224, RSPO2, and AZD5363.

### T5224, RSPO2, and AZD5363 induce apoptosis of FPA cells

**Figure 6:**
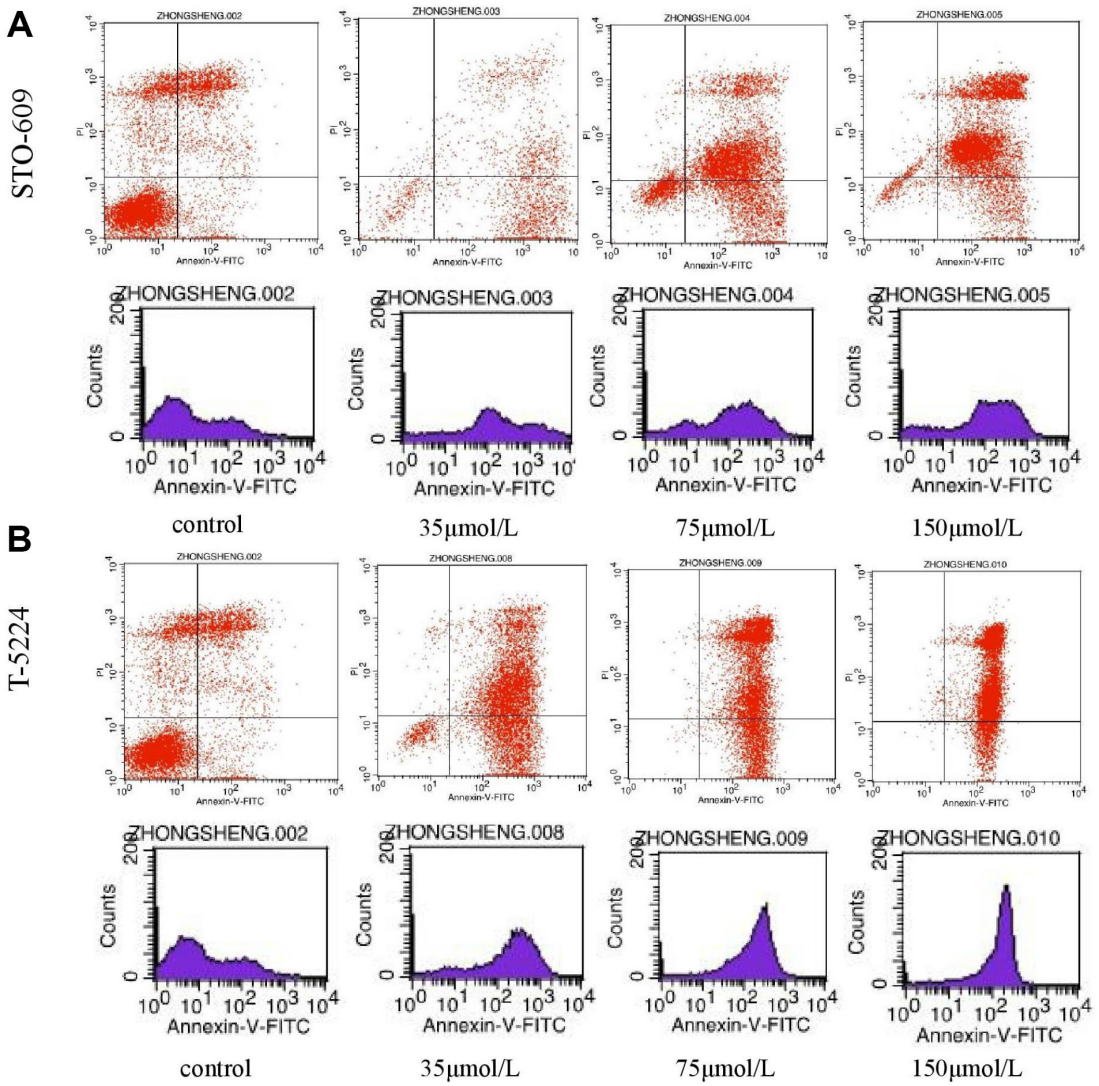
The distribution of cells in apoptosis with different doses of (**A**) STO-609 and (**B**) T5224.

**Figure 7:**
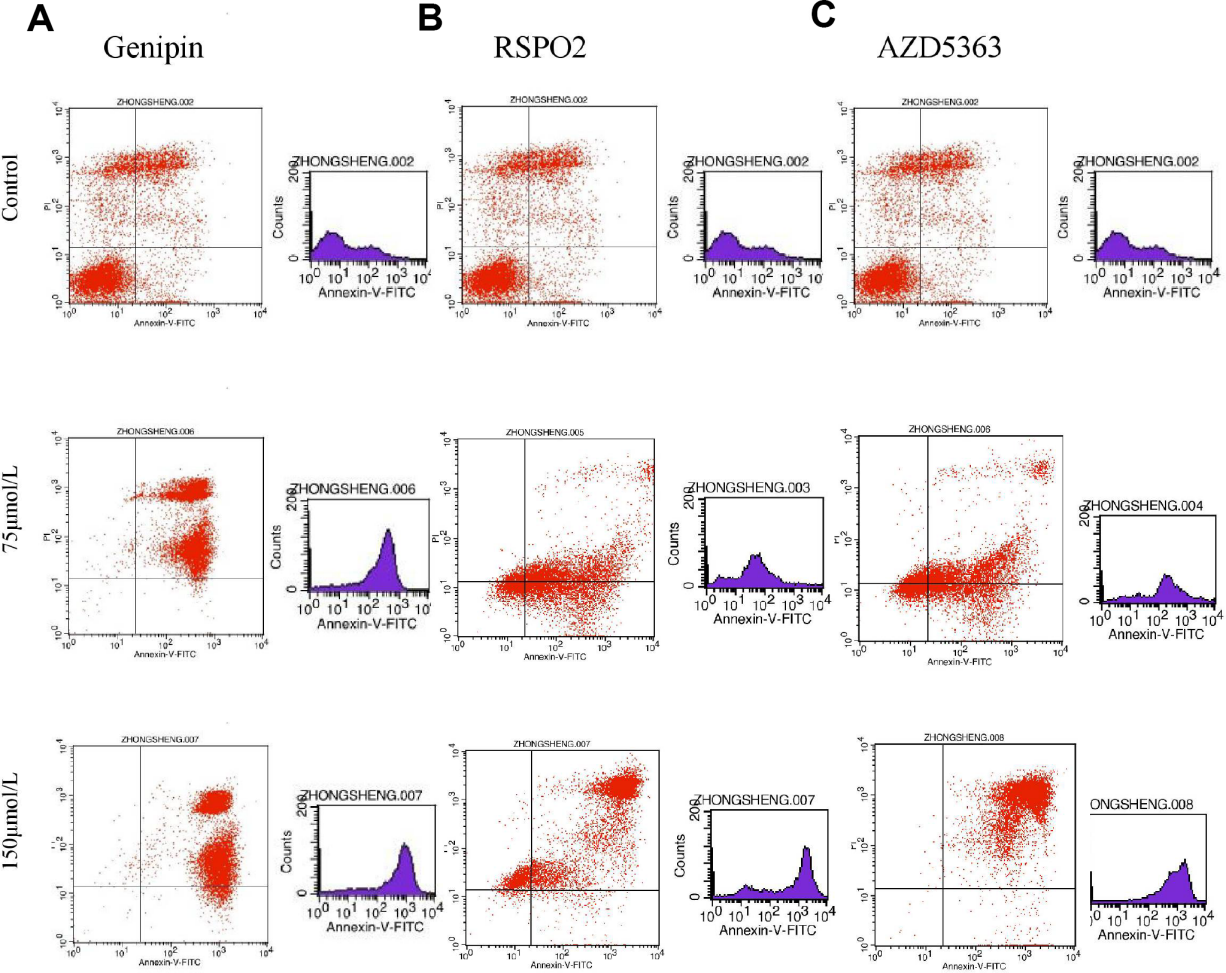
The distribution of cells in apoptosis with different dose of (**A**) Genipin, (**B**) RSPO2, and (**C**) AZD5363.

Flow cytometry of FPA cells and controls treated with different doses of drugs for 48 h allowed us to measure the percentages of normal, necrotic, late apoptotic, and early apoptotic cells. For controls, the respective percentages were 57.35%, 16.04%, 21.33%, and 5.28%. On the other hand, for T-5224-treated cells they were 5.69%, 1.08%, 59.74%, and 33.49% in low-dose group (10 μmol/L); 0.36%, 0.55%, 70.23%, and 28.86% in the intermediate-dose group (20 μmol/L); and 0.16%, 0.91%, 83.13%, and 15.8% in the high-dose group (40 μmol/L) (Figure 6A). For STO-609-treated cells, the numbers were 13.37%, 1.73%, 34.72%, and 50.17% in the low-dose group (35 μmol/L); 12.29%, 2.93%, 60.1%, and 24.68% in the middle-dose group (75 μmol/L); and 4.04%, 3.01%, 79.71%, and 13.24% in the high-dose group (150 μmol/L) (Figure 6B). The relevant numbers for Genipin were 0.23%, 1.08%, 97.28%, 1.41% in low dose group (75 μmol/L) and 0.22%, 0.51%, 90.53%, 8.74% in high dose group (150 μmol/L) (Figure 7A). For RSPO2-treated cells, the percentages were 7.19%, 4.15%, 48.04%, and 40.62% in the low-dose group (75 μmol/L), and 1.72%, 6.59%, 90.72%, and 0.97% in the high-dose group (150 μmol/L) (Figure 7B). For AZD5363-treated cells, the percentages were 6.91%, 3.75%, 52.22%, and 37.12% in the low-dose group (75 μmol/L), and 0.24%, 0.71%, 97.32%, and 1.73% in the high-dose group (150 μmol/L) (Figure 7C). We noticed that normal cells were predominant in the control group while apoptotic cells were predominant in the presence of T5224, RSPO2, or AZD5363 treatment. Compared to STO-609-treated cells, there was a greater percentage of apoptotic cells in the T5224, Genipin, RSPO2, and AZD5363-treated groups, even when using the same dose for all drugs.

## DISCUSSION

Many FPA patients present reduced quality of life, persistent morbidity, and slightly increased mortality in spite of receiving current therapies [4]. In the present study, we analyzed the gene expression profiles of 21 FPA samples and 14 normal samples from mRNA microarray datasets GSE2175, GSE26966, GSE36314, and GSE37153 in the GEO database. And a total of 19,943, 4635, 6472, and 2020 DEGs were identified respectively from those four datasets. There were 178 “mutual DEGs” identified by performing Venn plot among those four datasets.

GO analysis of abnormally expressed genes showed that upregulated genes were mainly associated with biological processes relevant to mitotic proliferation such as cell division, spindle formation, microtubule polymerization, protein binding, and ATP binding, which may explain the fast multiplication of cancer cells. Downregulated genes were primarily involved in biological processes underlying cell communication and signaling, including ion transport, cell junction and plasma membrane formation, calcium-dependent protein binding and calcium ion binding. Our results agree with a previous study showing that over-representation of genes can modify the course of the cell cycle, cell development, and cell differentiation/proliferation in pathologic adenoma cells [11]. Several studies have also shown that cell membrane ion channels, especially potassium channels, participate in cell signal transduction, proliferation, apoptosis, and regulation of gene expression in tumors [12]. Furthermore, the KEGG analyses we conducted here revealed that the mutual DEGs were mainly involved in the cell cycle, the p53 signaling pathway, oocyte meiosis, nicotine addiction, GABAergic synapse, and morphine addiction.

The levels of some cell-cycle regulators (p16, pRB protein, and cyclin D1) can predict the occurrence and proliferation of FPA [13]. The P53 signaling pathway is involved in cell cycle arrest, apoptosis, senescence, DNA repair, and changes in metabolism [14]. Thus, it is not surprising that P53 mutations are the most common in malignant tumors. The inhibition of p53 caused by c-Jun upregulation promotes FPA invasion [15]. Also, morphine promotes tumor growth by inhibiting apoptosis and promoting angiogenesis and migration of tumor cells [16]. Similarly, tobacco compounds have long been known to promote cell proliferation [17], suggesting that smoking may increase the probability of developing FPA.

c-Fos, one of the components of the activator protein-1 (AP-1) transcription factors, is hyper-activated in tumorigenesis and promotes cancer cell invasion and proliferation [18–19] for various types of cancer (Table 4) [18–20]. Injection treatment with FGFR1 inhibitor AZD4547 decreases the number and surface area of metastatic lung nodules and parenchyma in mice [21], highlighting AZD4547 as a potential treatment for other types of cancer. Similarly, T5224 inhibits AP-1, c-Fos, and therefore FGFR1, which suggests that it might exert anti-FPA effects.

**Table 4:** Hub genes and related cancers.

**Hub genes**	**Related cancers**
c-Fos	breast cancer, osteosarcoma, endometrial carcinoma, bladder cancer, prostate cancer, hepatoma cancer, et al.
WNT5A	prostate cancer, melanoma, gastric carcinoma, breast cancer, prostate cancer, lung metastasis of sarcoma cells, et al.
NCAM1	ovarian carcinoma, gastric cancer, melanoma, Wilms tumor (WT), et al.
JUP	colorectal cancer, oral intraepithelial neoplasms, breast cancer, serous ovarian cancer, testicular cancer, et al.
AKT3	glioma, breast cancer, leukemia, colon cancer and prostate cancer, et al.
ADCY1	esophageal carcinoma, pancreatic cancer, rectal adenocarcinoma, et al.

WNT5A promotes malignant progression in tumor cells [22–23] and is overexpressed in many types of cancer [22–24] (Table 4). RSPO2 inhibits tumor cell migration [49], implying it might also have therapeutic effects in FPA.

NCAM1 is a neural cell adhesion molecule that promotes cell-cell and cell-matrix interactions during development and cellular differentiation [25]. NCAM1 promotes other normal cellular processes [26] and genes in the underlying pathways are more likely to be deregulated in tumors that have migrated to lymph nodes, especially basal-like tumors associated with poor prognosis [25]. NCAM1 is also deregulated in other types of cancer (Table 4) [27], which suggests it might serve as a prognostic biomarker and therapeutic target for FPA [28–29].

The junction plakoglobin gene (JUP) is a desmosomal anchor protein gene, whose normal functioning is necessary for having healthy inter-cellular junctions and microtubules [30–31]. JUP is abnormally expressed in various diseases, including cancer (Table 4) [30–33], and its overexpression promotes metastasis and primary site recurrence in squamous cell carcinoma [34–35]; thus, JUP levels might also inform on the recurrence of FPA.

Protein kinase AKT3 promotes progression, metastasis, and drug resistance in various types of cancer (Table 4) [36–39]. AZD5363, a Akt3 inhibitor, inhibits proliferation in prostate cancer [50], which suggests it could also have therapeutic effects in in FPA.

ADCY1, adenylate cyclase 1, catalyzes the synthesis of cAMP [40–41] and was found to be dysregulated in rectal adenocarcinoma (RAC) and other cancers (Table 4) [40, 42]. In addition, ADCY1 overexpression promotes multi drug-resistant esophageal carcinoma-1 [43]. Fortunately, ADCY1 target drugs improve prognosis for esophageal carcinoma patients, which suggests that these drugs may also help FPA patients [43–44].

In this study, cFos, NCAM1, JUP, AKT3 ADCY1, CCND2, PPP2R5A, CAMK2G, ATP2A2, and MAD2L1 as well as hub genes were shown to be dysregulated in FPA and may serve as therapeutic targets or prognostic and diagnostic makers.

We used qRT-PCR to measure cFos, WNT5A, NCAM1, JUP, AKT3, and ADCY1 levels in normal pituitary cells (R1200) and FPA cell lines (GT1-1, GH3). We found that the expression of cFos, NCAM1, AKT3, and ADCY1 was lower in normal pituitary cells than in FPA cells (P < 0.05) while WNT5A and JUP levels were higher. This means that high expression of cFos, NCAM1, AKT3, and ADCY1 promotes tumorigenesis while high levels of AKT3 and ADCY1 inhibits it.

We used MTT and colony-formation assays to evaluate the effects of T5224, RSPO2, and AZD5363 on FPA. For cell lines GT-1 and GH3, cell viability correlated negatively with T5224, RSPO2, AZD5363, STO-609, and Genipin treatments in a dose-dependent manner. Furthermore, the ratio of T5224 groups dropped more significantly than the rest of groups in GH1-1 cell lines, including RSPO2 and AZD5363 groups. That indicates that T5224, RSPO2, and AZD5363 have therapeutic effects on FPA cells and protective effects on normal pituitary cells. Similarly, colony-forming assays showed that the number and size of clonogenicities in the drug groups were remarkably smaller than in controls and correlating negatively with treatment in a dose-dependent manner, with T5224, RSPO2, and AZD5363 yielding the smallest clonogenicities, in agreement with the results of our MTT assays.

Our flow cytometry experiments on FPA cells treated with different doses of T5224, RSPO2, AZD5363, STO-609, or Genipin for 48 h, showed that apoptosis correlated positively with treatment dose in all five drug groups compared to controls, suggesting beneficial effects from such drugs in the treatment of FPA.

Considering previous studies, the results of our *in vitro* study here indicate that T5224 exerts anti- FPA effects specifically by inhibiting cFos pathways and that RSPO2 does so by inhibiting Wnt5A while AZD5363 inhibits Akt3. Further studies *in vivo* should be conducted to test the therapeutic effects we uncovered here in more clinically-relevant systems.

## MATERIALS AND METHODS

### Microarray data

The gene expression profiles of GSE2175, GSE26966, GSE36314, and GSE37153 were obtained from the GEO database (https://www.ncbi.nlm.nih.gov/geo). The corresponding profiles were provided on platform GPL96 (GSE2175), GPL570 (GSE26966), GPL8300 (GSE36314), and GPL6480 (GSE37153) [45–48]. The GSE2175 contained one FPA samples and three normal samples, 14 FPA tissues and nine normal samples in the GSE26966. The GSE36314 provided four FPA samples and three normal pituitary tissues, and GSE37153 consisted of two FPA samples and one normal sample.

### Identification of DEGs

Analyses of the raw data were carried out using GeneSpring software (version 11.5, Agilent, USA) for four groups of DEGs to fit four respective gene expression profiles. The category of each data set was derived from hierarchical clustering. Group FPA and normal tissues were identified. The probe quality control in GeneSpring was limited by virtue of principal component analysis (PCA), and probes with intensity values below the 20^th^ percentile were filtered out using the “filter probesets by expression” option. Then, the DEGs were identified using classical *t* test with P value cutoff of < 0.05 and a change ≥ two fold. We also computed Venn diagrams for each DEG (http://bioinformatics.psb.ugent.be/webtools/Venn/).

### Gene ontology and pathway enrichment analysis of DEGs

The DAVID database (Database for Annotation, Visualization and Integrated Discovery, https://david.ncifcrf.gov/) provides a comprehensive annotation tools to understand the biological meaning underlying plenty of genes. GO (Gene Ontology) is a useful method for exposing biological process, molecular function, and cell component of genes. KEGG (Kyoto encyclopedia of Genes and Genomes) is a base for gene function analysis and genomic information linking. We performed GO and KEGG pathway enrichment analyses using DAVID for functional analyses of DEGs.

### PPI network construction and modules selection

We used the online database STRING (Search Tool for Retrieval of Interacting Genes, https://string.embl.de/) for PPI (Protein-Protein interaction) analysis. Then, we used Cytoscape software to screen hub genes and modules with MCODE (Molecular Complex Detection). Finally, we performed function and pathway enrichment analyses of DEGs in modules.

### Cell lines

Normal pituitary cells (R1200) and FPA cells (GT1-1 and GH3) were received from the ATCC (American Type Culture Collection). Those cell lines were cultured in Dulbecco’s modified Eagle’s medium (DMEM, Hyclone, USA) supplemented with 10% fetal bovine serum (FBS, Gibco, USA). The cell cultures were maintained at 5% CO_2_ and 95% air at 37 °C.

### Real-time quantitative reverse transcription PCR

To verify the expression of cFos, WNT5A, NCAM1, JUP, AKT3, and ADCY1 in FPA cell lines and normal human pituitary cells, we used FastStart Universal SYBR Green Master (ROX) (Roche Diagnostics) to perform qRT-PCR in a CFX96 Real-Time System (Bio-Red) according to the manufacturer’s instructions. Expression levels were normalized to glyceraldehyde-3-phosphate dehydrogenase (GAPDH). The 2^-ΔΔCt^ method was used for qRT-PCR data analysis. The primers of genes were enumerated as followed: cFos sense, 5′-CCTCTCCATGCAGGAGTTAAGA-3′; cFos anti-sense, 5′-GGTCTCGGGTCCTTGATTTTCT-3′; WNT 5A sense, 5′-TACTGCGGTGGAGCAAGAAG-3′; WNT5A anti-sense, 5′-CATCTGCGCTTGACGGAGA G-3′; NCAM1 sense, 5′-AGCCCATCAATAAGGGAG GG-3′; NCAM1 anti-sense, 5′-ACCTGACACCCGTTT TAGCTG-3′; JUP sense, 5′-TACTGCGGTGGAGCAA GAAG-3′; JUP anti-sense, 5′-CATCTGCGCTTGACG GAGAG-3′; ADCY1 sense, 5′-TACTGCGGTGGAGC AAGAAG-3′; ADCY1 anti-sense, 5′-CATCTGCGCTT GACGGAGAG-3′.

### MTT assay

The FPA cells (GT1-1, GH3) were plated into 96-well culture plate with a density of 500 cells/well, and were treated with different doses of T-5224, RSPO2, and AZD5363, respectively, as well as STO-609 (CaMKK inhibitor) and Genipin (aglycone derived from the iridoid glycoside), both of which protect against several types of tumors, including brain tumors. We used MTT (Sigma, St. Louis, Missouri, USA) dissolved in PBS (5 mg/ml) to measure the viability of cells. On the day of measurement, the medium was replaced on fresh DMEM supplemented with 10% FBS and diluted MTT (1:10, 10% MTT), and incubated for 3.5 h at 37 °C. Then, the incubation medium was removed and formazan crystals were dissolved in 200 μl solution of DMSO. We used an ELx800 absorbance microplate reader (BioTek Instruments, VT, USA) to quantify the MTT reduction by measuring light absorbance at 570 nm. Each test was repeated four times.

### Colony-forming assay

FPA cells (GT1-1, GH3) were seeded in Petri dishes with a density of 50 cells/cm^2^. After 24 h in culture, the cells were treated with different doses of STO-609, Genipin, T-5224, RSPO2, and AZD5363, respectively. After 10 days of growth *in vitro*, colonies were counted and described according to Franken et al. Then, colonies were rinsed with PBS, fixed in 4% paraformaldehyde, stained with 5% crystal violet for 0.5 h, and rinsed twice with water.

### Flow cytometry

The FPA cells (GT1-1) in the log growth phase were seeded into 6-well plates with a density of 2 × 10^5^ cells/well and treated with different doses of STO-609, Genipin, T-5224, RSPO2 and AZD5363. After 48 h of culturing, the cells were harvested using accutase detachment solution (Sigma Aldrich, USA). Annexin-V-FITC/PI labeling was conducted according to the manufactures’ instruction. a flow cytometer was used to count stained cells with the FACSDiva Version 6.2.

### Statistics

All statistical data analyses were carried out using SPSS 18.0 (SPSS Inc., Chicago, Illinois, USA), namely *t* tests for independent samples with P values < 0.05.

## Supplementary Material

Supplementary Table
